# Supplementary material for: Resistin is a risk factor for all-cause mortality in elderly Finnish population: A prospective study in the OPERA cohort
Source: PLoS One. 2021 Mar 2;16(3):e0248015. doi: 10.1371/journal.pone.0248015 (PMC7924753; doi:10.1371/journal.pone.0248015)
Supplement: Attachment — Submitted filename: Response to Reviewers..docx [file pone.0248015.s001.docx]

1.) Please ensure that your manuscript meets PLOS ONE's style requirements, including those for file naming. The PLOS ONE style templates can be found at

<https://journals.plos.org/plosone/s/file?id=wjVg/PLOSOne_formatting_sample_main_body.pdf> and

<https://journals.plos.org/plosone/s/file?id=ba62/PLOSOne_formatting_sample_title_authors_affiliations.pdf>

PLOS ONE’s style requirements have been checked and the necessary changes have been made.

 2.) Please include additional information regarding the survey or questionnaire used in the study and ensure that you have provided sufficient details that others could replicate the analyses. For instance, if you developed a questionnaire as part of this study and it is not under a copyright more restrictive than CC-BY, please include a copy, in both the original language and English, as Supporting Information.

The questionnaires used in the study are well known and are described in the revised manuscript. No new questionnaires were created.

3.) We note that you have indicated that data from this study are available upon request. PLOS only allows data to be available upon request if there are legal or ethical restrictions on sharing data publicly. For information on unacceptable data access restrictions, please see <http://journals.plos.org/plosone/s/data-availability#loc-unacceptable-data-access-restrictions>.

In your revised cover letter, please address the following prompts:

a) If there are ethical or legal restrictions on sharing a de-identified data set, please explain them in detail (e.g., data contain potentially identifying or sensitive patient information) and who has imposed them (e.g., an ethics committee). Please also provide contact information for a data access committee, ethics committee, or other institutional body to which data requests may be sent.

Data cannot be shared publicly because of privacy policy. Data are available from the Oulu University Institutional Data Access for researchers who meet the criteria for access to confidential data. Contact information for the Oulu University Institutional Data Access committee: olavi.ukkola@oulu.fi, tel. +358400944539

b) If there are no restrictions, please upload the minimal anonymized data set necessary to replicate your study findings as either Supporting Information files or to a stable, public repository and provide us with the relevant URLs, DOIs, or accession numbers. Please see <http://www.bmj.com/content/340/bmj.c181.long> for guidelines on how to de-identify and prepare clinical data for publication. For a list of acceptable repositories, please see <http://journals.plos.org/plosone/s/data-availability#loc-recommended-repositories>.

We will update your Data Availability statement on your behalf to reflect the information you provide.

4) Thank you for stating the following financial disclosure:

 [NO. The funders had no role in study design, data collection and analysis, decision to
publish, or preparation of the manuscript.].

At this time, please address the following queries:

a. Please clarify the sources of funding (financial or material support) for your study. List the grants or organizations that supported your study, including funding received from your institution.
b. State what role the funders took in the study. If the funders had no role in your study, please state: “The funders had no role in study design, data collection and analysis, decision to publish, or preparation of the manuscript.”
c. If any authors received a salary from any of your funders, please state which authors and which funders.
d. If you did not receive any funding for this study, please state: “The authors received no specific funding for this work.”

The authors received no specific funding for this work.

Please include your amended statements within your cover letter; we will change the online submission form on your behalf.

**Reviewer comments**

Reviewer #1: Comments to the paper entitled "Resistin is a risk factor for all-cause mortality in elderly Finnish population: a prospective study in the OPERA cohort"

The authors evaluated a total of 599 Finnish elderly population with a mean follow-up of 5.6 years. They found that resistin is associated with prospective all-cause mortality in this general population of elderly.

The paper should be revised to a major extent, as:

1. No hazard ratio (HR) was provided for the second tertile in the unadjusted model, and all HRs were missing in the adjusted model. They should be listed in a separate TABLE or FOREST PLOT. Moreover, the authors did not adjust for blood pressure as continuous variable(s) in their analysis. Since blood pressure is a significant predictor of cardiovascular and all-cause mortality, it should be added in the model as covariate(s). The necessity was further emphasized in line 280-283, where the authors citied the study by Muse et al. In that study systolic blood pressure was also evaluated by replacing hypertension as sensitivity analysis.

Thank you for the suggestions. We have now included all the HRs in a separate table (Table 2). We have also replaced “hypertension” with systolic blood pressure as a covariate in the multivariate analysis. Unlike, Muse et al., our study population did not demonstrate increasing systolic blood pressure across increasing resistin quartiles/tertiles (Table 1). Interestingly, neither systolic blood pressure (HR: 0.990, 95% CI: 0.978-1.001, p=0.073) or diastolic blood pressure significantly predicted all-cause mortality within our study. In fact, diastolic blood pressure seemed to even be a protective factor (HR: 0.967 95% CI: 0.945-0.990, p=0.005).

2. Resistin should be log-transformed if the distribution is, as stated in line 86-87, "average resistin concentration was 11.0 ± 5.05 ng/ml and varied from 2.94 to 43.1 ng/ml". Its association with mortality, as revealed in the figure, also supports such transformation that the survival of 1st and 2nd resistin tertile was not of significant difference when inspected visually. Cutoff for resistin, albeit not a must, is also welcomed if provided.

That is correct. We have now included resistin as a log-transformed variable in our analyses. We also analyzed receiver operating characteristic (ROC) curve (Figure 1) to determine optimal cutoff for resistin (12.88 ng/ml). Additional survival analyses were conducted by dividing the study population into two groups based on the cutoff resistin concentration (≥ 12.88 ng/ml vs < 12.88 ng/ml). Those who were above the cutoff had over 3-fold higher risk for all-cause mortality compared to those below the cutoff (p<0.001). The results are presented in the Table 2.

3. Table 1 and 2 were redundant when table 3 presented. They should be removed.

Table 1 and 2 have been removed and the baseline characteristic of our study population are currently displayed only in Table 3 (Table 1 in the revised manuscript).

4. The figure should be improved to include p value of log-rank test at the upper-left part and number at risk at the lower part of the figure.

Figure 1 (Figure 2 in the revised manuscript) has been improved as per your suggestion to include the p-value of the log-rank test as well as the number at risk information.

5. Amount of cigarette /alcohol should be described carefully as the distribution of them was largely skewed. The alternative, the proportion of current/history of smoking/heavy drinking, is a better replacement if never-smokers and occasional drinkers constitute a great proportion of the whole.

That is a valid notion. The alcohol consumption and smoking habits are now being presented (Table 1) and analyzed as categorical variables:

Alcohol consumption

1. Never
2. Not anymore
3. Occasionally (once or less per week)
4. More than once a week
5. Almost daily

Smoking habits

1. Never
2. Current smoker
3. Not anymore
4. Irregularly

6. The discussion part should be re-categorized into three major parts, 1. comparison and contrast with previous population study, 2. mechanisms involved in the association, and 3, clinical perspective. In fact, more than half of all your references (Ref #1-8, 10-13, 15, 16, 18, 20, 27, 34, 39-41) came from the first reference. Although citing these papers could enrich your argument on the mechanistic prospective for enabling such association between resistin and all-cause mortality, these arguments and related references should be abbreviated. Only the most important papers relevant to the current findings should be citied.

Thank you for pointing out these issues. The discussion part has now been completely re-organized to make it easier to follow, the essential arguments have now been presented in a more concise manner and unnecessary citations have been removed. The discussion now consists of the following sections:

1. Comparison and contrast with previous studies (paragraphs 2-3, lines 219-245)
2. Mechanisms involved in the association (paragraphs 4-6, lines 246-286)
3. Clinical perspective (paragraph 7, lines 287-298)

   Some minor typing errors/suggestions:
   line 22 "monoculear" should be "mononuclear"
   line 57 a comma "," should be added after ref [11]
   line 134 a comma "," should be added after "total".
   line 136-137 the concentration starting with (1.63...) should follow HDL but not cholesterol.
   line 137 "represented" should be "presented"
   line 238 "inceased" should be "increased"
   line 289 "spesific" should be "specific"

These typing errors have been corrected.

Reviewer #2: In this manuscript, the author investigated that whether resistin predicts mortality among elderly Finnish people. The study population consisted of 599 elderly (71.7 ± 5.4 years) patients and the follow-up was approximately six years. After the follow-up, resistin was a significant risk factor for all-cause mortality (HR 3.02, 95 % CI: 1.64-5.56, p<0.001) even after adjusting for various covariates such as age, sex, hypertension, diabetes, hsCRP, physical activity, smoking and alcohol consumption. The result indicated that resistin is a significant risk factor for all-cause mortality among elderly Finnish subjects, independent from traditional cardiovascular risk factors. However, there are several issues need to be considered.
1. How about the distribution of resistin in the population? Do you need to use the log transformed value.

The distribution of resistin, indeed, is heavily skewed which is why we have now included resistin as log-transformed in all of our analyses.

2. This study demonstrated that resistin was a significant risk factor for all-cause mortality when analyzed as a continuous variable as well as when the highest and lowest tertile were compared. It would be better if you do Receiver operator characteristic (ROC) curve analysis for serum resistin to determine the possible cutoff value for resistin.

After performing ROC curve analysis, we determined the optimal cutoff for resistin to be 12.88 ng/ml. We also performed additional survival analyses by dividing the study population in two groups: 1) resistin ≥ 12.88 ng/ml and 2) resistin < 12.88 ng/ml. The results of these survival analyses are being presented in Table 2.

3. This study demonstrated that resistin was a significant risk factor for all-cause mortality. How about the CV mortality? Do you have the related data?

Unfortunately, we do not have the data related to cardiovascular events/mortality. Previous studies have reported somewhat conflicting results on the role of resistin in predicting cardiovascular events. Muse et al. (2015), for instance, reported that resistin significantly predicted cardiovascular events independent from various covariates (including inflammatory markers) whereas Gencer et al. (2016) reported that the significance was lost after adjusting for inflammatory status. Our results indicate that resistin predicts all-cause mortality independent from inflammatory status (hsCRP), and therefore it would have been interesting to see whether or not this effect persists with regards to cardiovascular events/mortality.

4. I wonder whether or not the highly increased total mortality risk was modulated by synergistic interaction with various demographic and clinical features. To this purpose, mortality risk should be compared across subgroups in a multivariable model considering general confounders, such as sex, age at recruitment, smoking habits and so on.

Thank you for the great notion. We performed additional survival analyses and adjusted the multivariate model for the covariates used in the general multivariate analyses (age, sex, systolic blood pressure, smoking habits, alcohol consumption, medications [antihypertensive, lipid-lowering, glucose-lowering], hsCRP and leisure time physical activity). The following subgroups were analyzed:

1. Age tertiles
2. Gender
3. Hypertensive vs non-hypertensive
4. Diabetics vs non-diabetics
5. Never smokers vs current smoker/history of smoking
6. Alcohol consumption (never vs yes)

The results showed that the resistin remained a significant predictive factor for all-cause mortality only in the following subgroups:

1. Age (only the highest tertile), p=0.001
2. Hypertensives, p=0.038
3. Diabetics, p=0.002
4. Alcohol consumption (those who used/had used), p=0.006

These results have now been mentioned briefly in the results section (lines 189-192).

5. In Figure 1, the accurate resistin concentration in different tertiles and the P value should be illusterated.

Figure 1 (Figure 2 in the revised manuscript) has now been improved to include the resistin concentrations for each tertile, the p-value of the log-rank test as well as the number at risk information.

6. Univariate Cox-regression analysis and multivariate Cox regression analysis should be illustrated in Table in different models. Glucose-lowering, anti-hypertensive, antidyslipidemic treatments should be adjusted in multivariate Cox regression analysis.

We have now created a new Table 2 (Table 1 and 2 in the original manuscript have been removed) that presents the hazard ratios for resistin in univariate and multivariate analyses. Antihypertensive, lipid-lowering and glucose-lowering medications have been included in the multivariate analyses as per your request.

7. What’s the device you used in this study to measure blood pressure? And how many times you measured to define hypertension? What’s the definition of diabetes? You should introduce EQ-5D and MMSE briefly in the Methods.

The following adjustment was made in the methods section (lines 84-91) to clarify the blood pressure measurements as well as the definition of diabetes: “Blood pressure was measured according to the recommendations of the American Society of Hypertension [17] in a sitting position from the right arm with an oscillometric device (Dinamap® model 18465X, Criticon Ltd., Ascot, UK) after an overnight fast and after a 10-15-minute rest. Three measurements were made at 1-minute intervals and the means of the last two were used in the analyses. Hypertension was defined as blood pressure over 140/90 or current antihypertensive medication. Diabetes was defined as a known diabetes mellitus diagnosis (fasting plasma glucose of 7.0 mmol/l or more or 2-h plasma glucose of 11.1 mmol/l or more in the oral glucose tolerance test) at baseline visit.”

EQ-5D and MMSE were introduced in the methods section (lines 92-100) as follows: “The Mini-Mental State Examination (MMSE) is an easy-to-use tool for evaluating one’s memory and the processing of information. MMSE consists of simple questions and tasks, and correct answers accumulate points (the maximum being 30). The lower the score, the worse the memory function. Likewise, the EQ-5D is a standardized questionnaire for assessing one’s overall health status. It consists of two parts: descriptive and evaluation. In the evaluation part, the patients rate their overall health status using the visual analogue scale (ranging from 0 to 100), respectively. The scores of the EQ-5D evaluation part are presented in the current study.”

8. In line 126-129, you mentioned that :the discrimination abilities of resistin were assessed by the C-index by adding the following covariates into established model: age, hypertension, diabetes, hsCRP, physical activity and resistin. However, I did see any result about the C-index in this manuscript. Maybe you’d better add ROC curve in your manuscript.

That is a good idea. We have now presented the ROC curve as Figure 1. The statistical methods used to assess the discrimination abilities of resistin have been clarified in the methods section (lines 137-140).
